# Comparison of endoscopic ultrasound-guided fine-needle aspiration and biopsy with 22-gauge and 25-gauge needles for the “precision medicine” of pancreatic cancer: A retrospective study

**DOI:** 10.1097/MD.0000000000011096

**Published:** 2018-06-15

**Authors:** Naohiko Yoshizawa, Reiko Yamada, Takashi Sakuno, Hiroyuki Inoue, Hiroshi Miura, Toshifumi Takeuchi, Misaki Nakamura, Yasuhiko Hamada, Masaki Katsurahara, Kyosuke Tanaka, Noriyuki Horiki, Yoshiyuki Takei

**Affiliations:** a Department of Gastroenterology and Hepatology, Mie University Hospital; b Department of Endoscopy, Mie University Hospital, Tsu, Mie, Japan.

**Keywords:** 22-gauge needles, 25-gauge needles, endoscopic ultrasound-guided fine-needle aspiration and biopsy, immunohistochemical analysis, pancreatic ductal adenocarcinoma, precision medicine

## Abstract

We compared the sample volume of endoscopic ultrasound-guided fine-needle aspiration and biopsy (EUS-FNAB) specimens obtained by 22-gauge (22G) and 25-gauge (25G) needles, and the accuracy rate.

This was a retrospective study in a single tertiary referral center. We investigated 153 patients with pancreatic ductal adenocarcinoma (PDAC) who underwent diagnostic EUS-FNAB before neoadjuvant gemcitabine-based chemoradiotherapy between October 2006 and November 2015. We performed immunohistochemical (IHC) analysis of human equilibrative nucleoside transporter 1 using the remnant cell blocks following pathological PDAC diagnosis. We compared the sampling rate, accuracy rate, and success rate of IHC analysis between 22G and 25G.

There were 70 patients in the 22G group and 83 patients in the 25G group. The overall sampling rates on cytology and histology were 100% and 98.0%, respectively. The sampling rate did not differ between the 22G and 25G groups. The overall diagnostic accuracy rates on cytology and histology were 94.8% and 79.7%, respectively. The accuracy rates of 22G and 25G groups on cytology were 94.3% and 95.2%, respectively, whereas those on histology were 80.0% and 79.5%, respectively. The diagnostic accuracy on cytology and histology did not differ significantly between the 22G and 25G groups. Of 153 histology specimens, 69.3% of those with PDAC provided sufficient samples for IHC analysis. The success rate of IHC analysis did not differ significantly between the 22G (67.1%) and 25G (71.1%) groups (*P* = .60).

Both 22G and 25G provided a high diagnostic yield with equivalent accuracy rates on histology. EUS-FNAB specimens obtained using 22G or 25G can be equally adequate for IHC analysis and may be suitable for diagnostic examination. Further investigations such as EUS-FNAB needle design and novel cell block preparation are needed to obtain adequate samples for use in “precision medicine.”

## 1 Introduction

Pancreatic ductal adenocarcinoma (PDAC) is one of the most lethal malignancies. Median overall survival in patients with advanced PDAC who receive gemcitabine is <7 months. Even with the recent new combination chemotherapy regimens (e.g., oxaliplatin, irinotecan, fluorouracil, and leucovorin regimen or gemcitabine and albumin-bound paclitaxel regimen), the overall survival is <1 year.^[1–3]^ Therefore, combination with traditional cytotoxic therapies and personalized therapeutic approaches based on the genetic profile of each patient is desired in an effort to improve outcomes in the future.

The recent development of next-generation sequencing technologies is expected to make therapy by this novel approach feasible. Whole genome association studies have clarified the mutations associated with prognostic value^[4]^ and allowed the identification of patients who may respond to targeted therapies.^[5,6]^ There are several hurdles to this approach, such as the acquisition of adequate amount of tumor sample for molecular profiling in the clinical setting.^[7,8]^ For this novel therapy to be successful, the initially obtained specimen should contain viable tumor cells, not only for diagnosis but also for any potential therapeutic examinations.

Endoscopic ultrasound-guided fine-needle aspiration and biopsy (EUS-FNAB) can be a suitable method for obtaining such specimens. EUS-FNAB is considered as a safe and useful technique for the diagnosis of solid pancreatic tumors based on many reports evaluating the diagnostic yield. However, only a few reports have evaluated its ability to obtain a sufficient sample.

We reported that immunohistochemical (IHC) expression of human equilibrative nucleoside transporter 1 (hENT1) in pretreatment PDAC specimens obtained with EUS-FNAB was associated with the prognosis of patients with PDAC receiving gemcitabine-based chemoradiotherapy.^[9]^ In this study, we used remnant EUS-FNAB materials following cytological and/or histological diagnosis. Some of these samples might have been adequate as they were used before IHC analysis. In this situation, hENT1 IHC analysis was successfully performed in 68.4% cases of PDAC.

As for needle size, several reports have compared the diagnostic yield of 22-gauge needles (22G) and 25-gauge needles (25G) in facilitating the cytological diagnosis of PDAC.^[10,11]^ Generally, 22G may be suitable for pathological diagnosis because they collect larger sample volumes than 25G. Nevertheless, a recent report suggested that 25G are more sensitive than 22G for diagnosing pancreatic malignancy.^[12]^ It is suggested that 25G is easier for puncture, and result in fewer bloody and contaminated specimens than 22G.^[13,14]^

Hence, we aimed to evaluate the adequacy of sample quantity for “precision medicine” by comparing the success rates of IHC analysis in samples obtained by 22G and 25G.

## 2 Patients and methods

### 2.1 Study design

This was a retrospective study in a single tertiary referral center.

### 2.2 Participants

We investigated 153 patients with PDAC who underwent diagnostic EUS-FNAB before neoadjuvant gemcitabine-based chemoradiotherapy between October 2006 and November 2015. We compared the sampling, accuracy, and success rates of IHC analysis in samples obtained by 22G and 25G.

We obtained written informed consent for EUS-FNAB from all patients. The study protocol was approved by the Institutional Review Board for Human Research of Mie University Hospital.

### 2.3 EUS-FNAB procedure and diagnosis

A convex-array echo-endoscope (GF-UC240P and 260, Olympus, Tokyo, Japan) was used for EUS-FNAB. We punctured the pancreatic mass under endoscopic ultrasonographic guidance, after identifying the tumor using B-mode imaging, and confirming the absence of vessels in the target area. We used 2 types of needle: 22G (Echo tip, Wilson-Cook, Winston Salem, NC) and 25G (Expect, Boson-Scientific, Tokyo, Japan). The different needles were used according to their availability.

Cytologists immediately examined the specimen with rapid on-site evaluation using rapid staining (Diff-Quik stain; International Re-agents, Kobe, Japan) to verify that the obtained sample was sufficient. If the sample was insufficient, further punctures were performed. We confirmed the diagnosis of PDAC by cytological and/or histological analyses with EUS-FNAB specimen. The both cytological and pathological diagnoses were based on the review of all these materials by the cyto-pathologists. Thereafter, we retrospectively evaluated 153 stored cell blocks using IHC analysis for hENT1 expression as shown in our previous study.^[9]^ We defined the success group of IHC analysis using remnant cell blocks as the available group. In terms of cellularity of the cell block, remnant specimens included more than 50 lesional cells. IHC staining was performed using the labeled streptavidin–biotin peroxidase complex method with the Benchmark XT auto-immunostaining system (Ventana Japan, Tokyo, Japan). Rabbit polyclonal anti-SLC29A1 (ENT1) antibody (Medical and Biological Laboratories Co, Ltd, Nagoya, Japan) was used as the primary antibody.

Final diagnoses were made according to surgical histology and clinical follow-up for a minimum of 6 months.

### 2.4 Analysis

The primary outcome was the adequacy of the obtained sample volume using 22G and 25G determined according to the success rate of IHC analysis. The secondary outcome was the sampling and accuracy rates of procedures performed using 22G and 25G. The sampling rate of cytology was defined as the rate of obtaining pancreatic cells regardless of whether malignant cells could be confirmed. We defined the sampling rate of histology as the rate of obtainability of gray-whitish, worm-like tissue samples that were visible macroscopically. Diagnostic accuracy was defined as the rate of diagnosis as adenocarcinoma. Atypical and benign were defined as negative.

Statistical tests were performed using SAS University Edition (SAS Institute Inc, Cary, NC). We performed the Fisher exact test to compare categorical variables and the Wilcoxon rank-sum test to compare continuous variables. A *P* value < .05 was considered to represent a statistically significant difference.

## 3 Results

In total, 153 patients underwent EUS-FNAB: 70 patients in the 22G group and 83 patients in the 25G group. No statistically significant differences in the characteristics of the patients (age and sex), tumor location, tumor size, or resectability were found between both groups (Table 1). There was a significant difference between both groups (*P* = .003, Table 1) according to the International Union Cancer Control-T classification. However, there was no variable associated with diagnostic accuracy in the multivariate logistic regression analysis.

**Table 1:**
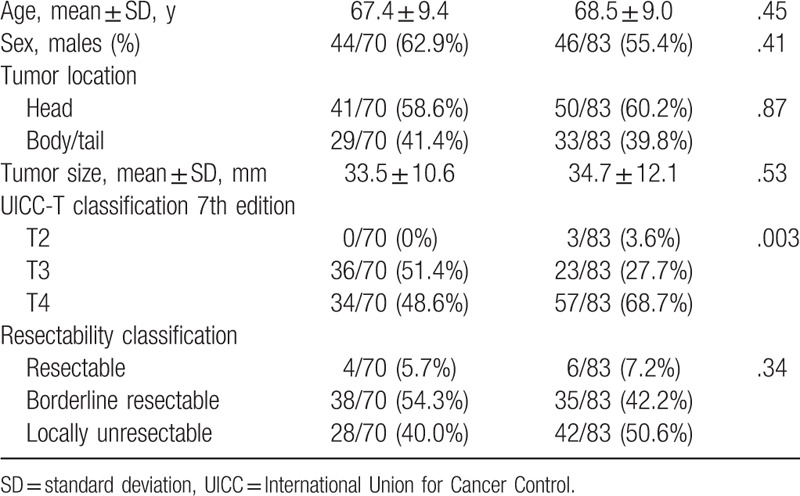
Patients’ characteristics.

The 22G and 25G groups had high sampling rates on cytology and histology with no difference in the sampling rate (Table 2). The overall sampling rates on cytology and histology were 100% (153/153) and 98.0% (150/153), respectively. The mean number of puncture with 25G was 2.54 times, which was significantly fewer than that of 22G: 3.09 times (*P* = .003). The sampling rates of the 22G and 25G on cytology were 100% (70/70) and 100% (83/83), and those on histology were 98.6% (68/70) and 97.6% (82/83).

**Table 2:**
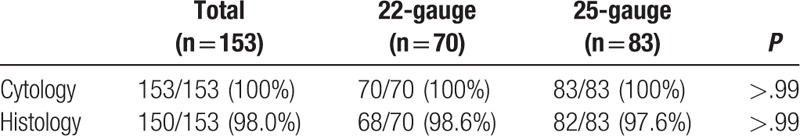
Sampling rate of 22- and 25-gauge on cytology/histology.

The diagnostic accuracy of cytology, histology, and combined analyses on both cytology and histology did not differ significantly between the 22G and 25G groups (Table 3). In addition, the combined analyses for 22G and 25G had a synergistic effect on cytology or histology alone. The overall diagnostic accuracy rates on cytology and histology were 94.8% (145/153) and 79.7% (122/153), respectively. The accuracy rate of biopsies performed using the 22G and 25G on cytology were 94.3% (66/70) and 95.2% (79/83), whereas those on histology were 80.0% (56/70) and 79.5% (66/83), respectively. The overall accuracy rate of combined analyses on cytology and histology was 96.7% (148/153). The accuracy rate of combined analyses of 22G and 25G was better than that on cytology or histology alone: 97.1% (68/70) for 22G and 96.4% (80/83) for 25G.

**Table 3:**
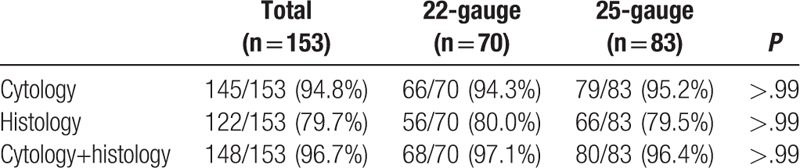
Accuracy of 22- and 25-gauge on cytology/histology.

Five cases could not be diagnosed using EUS-FNAB findings. The final diagnoses in these 5 cases were confirmed based on cytology of pancreatic juice obtained with endoscopic retrograde cholangiopancreatography (n = 1), surgical pathology (n = 1), and follow-up examination of tumor metastasis (n = 3).

We performed IHC analysis of hENT1 using remnant cell blocks following cytological and/or histological diagnosis of PDAC. The overall success rate of IHC analysis was 69.3% (106/153). The success rate of IHC analysis did not differ significantly between the 22G (67.1%, 47/70) and 25G (71.1%, 59/83) groups (*P* = .60) (Table 4).

**Table 4:**
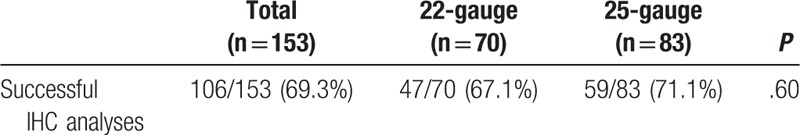
Success rate of immunohistochemical (IHC) analyses.

No significant difference was observed in factors other than age between the available and unavailable groups (Table 5). The mean age was 66.8 years in the available group and 70.8 years in the unavailable group (*P* = .012).

**Table 5:**
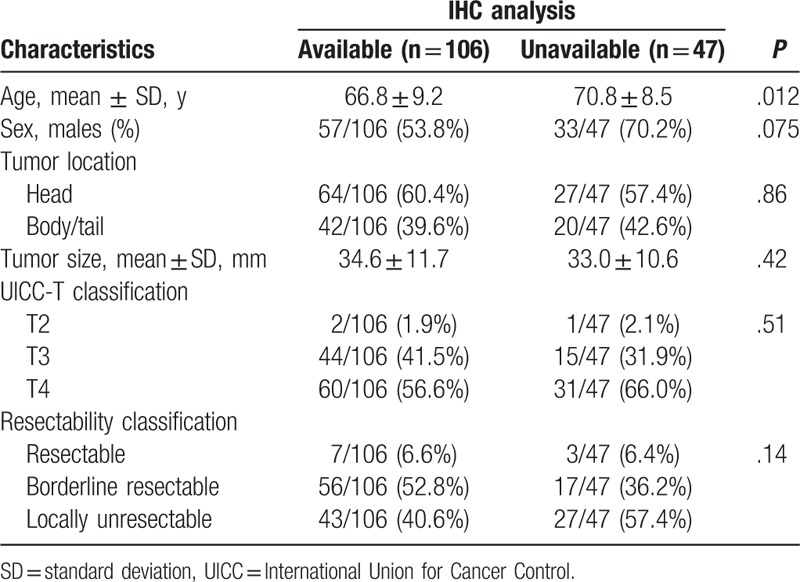
Patients’ characteristics according to immunohistochemical (IHC) analyses.

## 4 Discussion

We demonstrated that EUS-FNAB using both 22G and 25G have a high diagnostic yield. Furthermore, we evaluated the feasibility of IHC analysis for hENT1, and showed that both 22G and 25G performed well for sample acquisition. In our study, EUS-FNAB had a higher diagnostic yield than that reported in previous studies; the accuracy rates on cytology and histology were 94.8% and 79.7%, respectively. The accuracy rates on histology did not differ between 22G and 25G. In addition, the remnant specimens of 69.3% patients were available for IHC analyses, the rate of suitability for IHC analysis did not differ significantly between 22G and 25G.

Several studies showed the diagnostic accuracy of EUS-FNAB and compared the accuracy rates of 22G and 25G. In these studies, 25G showed a higher accuracy rate than 22G on cytology.^[10–12]^ The ease of puncture due to the flexibility of the thinner 25G might influence the accuracy of these needles.^[13]^ However, the accuracy rate of these different needle gauges on histology is controversial. Sakamoto et al reported that the accuracy rate on histology was significantly different between 22G (62.5%) and 25G (45.8%).^[10]^ By contrast, Kida et al^[15]^ reported that there were no differences between 22G (68.0%) and 25G (69.6%). Park et al^[16]^ also showed similar evidence: 22G (68.2%) and 25G (68.3%). In agreement with these 2 later reports, our results showed no difference between 22G and 25G (80.0% and 79.5%). However, our study showed higher diagnostic accuracy than the previous studies. We suggest that rapid on-site evaluation by pathologists increased the accuracy rate.

In addition to diagnosis, the remnant specimens of 69.3% patients were available for IHC analyses. Furthermore, the rate of suitability for IHC analysis did not differ significantly between 22G and 25G. We demonstrated that both 22G and 25G performed well for sample acquisition. Other than the present study, only a few reports have evaluated whether EUS-FNAB can be a reliable source of sufficient material for “precision medicine,” with special emphasis on needle size. Boone et al reported the correlation between SMAD4 expression in pretreatment PDAC tissues and the prognosis of PDAC patients. Their study design was similar to that of the present study, which used the remnant sample after diagnosis to evaluate SMAD4 protein: only 44.4% of samples could be analyzed.^[17]^ Navina et al evaluated the cellularity of EUS-FNAB material by scoring the number of lesional cells; only 12.4% of specimens had more than 100 cells, which was deemed to represent sufficient cellularity for “precision medicine.”^[18]^ From these results, it can be inferred that the success rate of “precision medicine” using EUS-FNAB samples obtained from PDAC specimens remained lower than that desired. In other reports, most samples used for molecular analysis, including next-generation sequencing, were obtained via surgery rather than EUS-FNAB. Specimens obtained via EUS-FNAB were deemed to contain poor-quality DNA due to the presence of intratumor heterogeneity and desmoplastic changes in PDAC.^[7,19]^

Recently, there are a few challenges to using next-generation sequencing on samples obtained via EUS-FNAB, including molecular aberrations.^[20–22]^ Young et al^[22]^ reported that next-generation sequencing on EUS-FNAB cell blocks yielded genomic profiles in all cases (17/17). Furthermore, new core biopsy needles have been introduced in clinical practice, which are expected to acquire more quantity of cells and to be more useful for “precision medicine.” Therefore, future studies should evaluate new EUS-FNAB needle designs. In addition, novel technologies with the potential to yield useful material from small sample volumes should be investigated to facilitate the development of personalized treatment approaches for patients with PDAC.

There are a few key limitations to the present study that should be discussed. First, this was a retrospective and single-center study. Therefore, the possibility of selection bias could not be excluded. Second, the adequacy rate of IHC was not high enough for clinical use in “precision medicine” applications. Therefore, the quality and quantity of samples obtained via EUS-FNAB should be assessed and further improved.

In conclusion, the results of this study showed that both 22G and 25G needles provided a high diagnostic yield and that EUS-FNAB specimen obtained using either 22G or 25G needles can be equally adequate for IHC analysis. It is envisaged that EUS-FNAB will be a reliable source of “precision medicine.”

## Author contributions

**Conceptualization:** Reiko Yamada.

**Data curation:** Naohiko Yoshizawa, Reiko Yamada, Takashi Sakuno, Hiroyuki Inoue, Hiroshi Miura, Toshifumi Takeuchi.

**Formal analysis:** Naohiko Yoshizawa.

**Funding acquisition:** Reiko Yamada.

**Investigation:** Naohiko Yoshizawa, Reiko Yamada, Takashi Sakuno, Hiroyuki Inoue.

**Methodology:** Reiko Yamada.

**Project administration:** Reiko Yamada.

**Resources:** Reiko Yamada, Takashi Sakuno, Hiroyuki Inoue, Hiroshi Miura, Toshifumi Takeuchi.

**Supervision:** Kyosuke Tanaka, Noriyuki Horiki, Yoshiyuki Takei.

**Validation:** Misaki Nakamura, Yasuhiko Hamada, Masaki Katsurahara.

**Writing – original draft:** Naohiko Yoshizawa, Reiko Yamada.

**Writing – review and editing:** Reiko Yamada.
